# 2-Amino-7-oxo-4,5,6,7-tetra­hydro-1-benzothio­phene-3-carbonitrile

**DOI:** 10.1107/S160053681003730X

**Published:** 2010-09-25

**Authors:** Mohamed Ziaulla, Afshan Banu, Noor Shahina Begum, Shridhar I. Panchamukhi, I. M. Khazi

**Affiliations:** a Department of Studies in Chemistry, Bangalore University, Bangalore 560 001, Karnataka, India; b Department of Chemistry, Karnatak University, Dharwad 580 003, India

## Abstract

In the title compound, C_9_H_8_N_2_OS, the benzothio­phene ring is substituted with amino, oxo and carbonitrile groups. The thio­phene ring is essentially planar (r.m.s. deviation = 0.0003 Å), while the cyclo­hexene ring is in a half-chair conformation. In the crystal, N—H⋯O hydrogen bonds generate chains of mol­ecules in a zigzag pattern along the *b* axis. Pairs of N—H⋯N hydrogen bonds form centrosymmetric head-to-head dimers about inversion centres, corresponding to an *R*_2_^2^(12) graph-set motif. In addition, rather weak N—H⋯S inter­actions are also present in the structure and the supra­molecular assembly is further consolidated by π–π stacking inter­actions between the benzothio­phene rings, disposed at a distance of 3.742 (3) Å.

## Related literature

For the preparation of the title compound, see: Shetty *et al.* (2009 ▶). For general background, see: Jordan (2003 ▶); Russell & Press (1996 ▶); Mery *et al.* (2002 ▶). For related structures, see: Akkurt *et al.* (2008 ▶); Harrison *et al.* (2006 ▶); Vasu *et al.* (2004 ▶). For Cremer–Pople puckering parameters, see: Cremer & Pople (1975 ▶). For hydrogen-bond graph-set nomenclature, see: Bernstein *et al.* (1995 ▶).
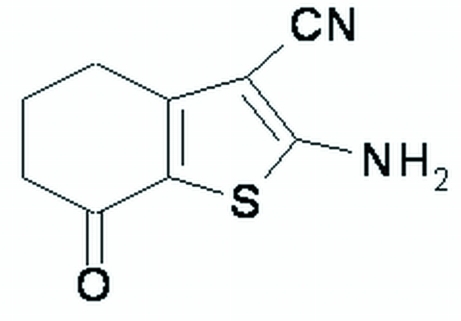


## Experimental

### 

#### Crystal data

C_9_H_8_N_2_OS
*M* *_r_* = 192.24
Monoclinic, 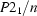

*a* = 7.2986 (3) Å
*b* = 8.7555 (3) Å
*c* = 14.7307 (6) Å
β = 94.151 (1)°
*V* = 938.87 (6) Å^3^
*Z* = 4
Mo *K*α radiation
μ = 0.30 mm^−1^
*T* = 296 K
0.20 × 0.18 × 0.18 mm

#### Data collection

Bruker SMART APEX CCD diffractometer
Absorption correction: multi-scan (*SADABS*; Bruker, 1998 ▶) *T*_min_ = 0.942, *T*_max_ = 0.947
6202 measured reflections
2058 independent reflections
1671 reflections with *I* > 2σ(*I*)
*R*_int_ = 0.020

#### Refinement

*R*[*F* ^2^ > 2σ(*F* ^2^)] = 0.043
*wR*(*F* ^2^) = 0.126
*S* = 1.02
2058 reflections
118 parameters
H-atom parameters constrained
Δρ_max_ = 0.70 e Å^−3^
Δρ_min_ = −0.34 e Å^−3^

### 

Data collection: *SMART* (Bruker, 1998 ▶); cell refinement: *SMART*; data reduction: *SAINT-Plus* (Bruker, 1998 ▶); program(s) used to solve structure: *SHELXS97* (Sheldrick, 2008 ▶); program(s) used to refine structure: *SHELXL97* (Sheldrick, 2008 ▶) and *PARST* (Nardelli, 1983 ▶); molecular graphics: *ORTEP-3* (Farrugia, 1997 ▶) and *CAMERON* (Watkin *et al.*, 1996 ▶); software used to prepare material for publication: *WinGX* (Farrugia, 1999 ▶).

## Supplementary Material

Crystal structure: contains datablocks global, I. DOI: 10.1107/S160053681003730X/pv2326sup1.cif

Structure factors: contains datablocks I. DOI: 10.1107/S160053681003730X/pv2326Isup2.hkl

Additional supplementary materials:  crystallographic information; 3D view; checkCIF report

## Figures and Tables

**Table 1:** Hydrogen-bond geometry (Å, °)

*D*—H⋯*A*	*D*—H	H⋯*A*	*D*⋯*A*	*D*—H⋯*A*
N1—H1*A*⋯N2^i^	0.86	2.19	3.038 (3)	169
N1—H1*B*⋯O1^ii^	0.86	2.10	2.903 (3)	156
N1—H1*B*⋯S1^ii^	0.86	3.02	3.482 (2)	116

Symmetry codes: (i) 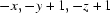; (ii) 
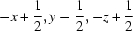
.

**Fig. 1.**
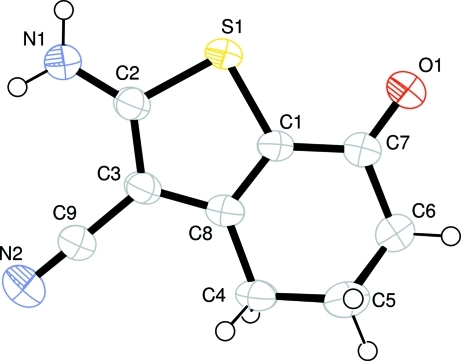
ORTEP (Farrugia, 1997) view of the title compound, showing 50% probability ellipsoids and the atom numbering scheme.

**Fig. 2.**
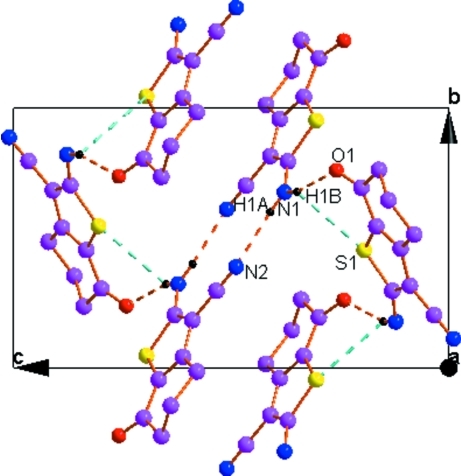
A unit cell packing of the title compound showing intermolecular hydrogen bonds with dotted lines. H atoms not involved in hydrogen bonding have been excluded.

## supplementary crystallographic information

### Comment

Benzothiophenes are important biologically active molecules. One of the most important drugs based on the benzothiophene system is Raloxifene, used for the prevention and treatment of osteoporosis in postmenopausal women (Jordan, 2003). Benzothiophenes are also luminescent components used in organic materials (Russell & Press, 1996). In addition, they are regarded as important units in liquid crystal research (Mery *et al.*, 2002). In this article, we report the structure of the title compound which has been synthesized in our laboratory.

In the title compound (Fig. 1), the thiophene ring is essentially planar while the cyclohexene ring is in a half-chair conformation; the atoms C5 and C6 deviate from the mean plane C1/C7/C4/C8 by 0.341 (3)and -0.233 (2) Å, respectively. The puckering parameters (Cremer & Pople, 1975) for the cyclohexene ring are: *Q*(2) = 0.3175 (3) Å, φ(2) = -17.96 (8)° and θ = 129.68 (7)°. In several benzothiophene derivatives the cyclohexyl ring adopts half-chair conformation, *e.g.*, (Akkurt *et al.*, 2008; Harrison *et al.*, 2006; Vasu *et al.*, 2004).

The N—H···O hydrogen bonds generate chains of molecules in a zigzag pattern along the *b*-axis. While the N—H···N hydrogen bonds form centrosymmetric, head-to-head dimers about inversion centers corresponding to graph set *R*^2^_2_(12) motif. In addition, rather weak N—H···S interactions are also present in the structure and the supramolecular assembly is further consolidated by π–π-stacking interactions between the benzothiophene rings; C—C disposed at a distance of 3.742 (3) Å.

The intermolecular interactions of the type N—H···O, N—H···N and N—H···S stabilize the crystal structure (Table 1). The N1—H1A···O1 hydrogen bonds generate chains of molecules in a zigzag pattern along the *b*-axis (Fig. 2). The N1—H1B···N2 hydrogen bonds on the other hand, form centrosymmetric, head-to-head dimers about inversion centers corresponding to graph set *R*^2^_2_(12) motif (Bernstein *et al.*, 1995) (Fig. 2). In addition, rather weak N1—H1B···S1 interactions are also present in the structure and the supramolecular assembly is further consolidated by π–π-stacking interactions between the benzothiophene rings; C—C disposed at a distance of 3.742 (3) Å.

### Experimental

The title compound was synthesized by following the procedure reported earlier (Shetty *et al.*, 2009).

### Refinement

The H atoms were placed at calculated positions in the riding model approximation with N—H = 0.86 and C—H = 0.97 Å, and *U*_iso_(H) = 1.2*U*_eq_(N/C).

### Crystal data

**Table:**

C_9_H_8_N_2_OS	*F*(000) = 400
*M**_r_* = 192.24	*D*_x_ = 1.360 Mg m^−^^3^
Monoclinic, *P*2_1_/*n*	Mo *K*α radiation, λ = 0.71073 Å
Hall symbol: -P 2yn	Cell parameters from 2058 reflections
*a* = 7.2986 (3) Å	θ = 2.7–27.0°
*b* = 8.7555 (3) Å	µ = 0.30 mm^−^^1^
*c* = 14.7307 (6) Å	*T* = 296 K
β = 94.151 (1)°	Block, yellow
*V* = 938.87 (6) Å^3^	0.20 × 0.18 × 0.18 mm
*Z* = 4	

### Data collection

**Table:**

Bruker SMART APEX CCD diffractometer	2058 independent reflections
Radiation source: Enhance (Mo) X-ray Source	1671 reflections with *I* > 2σ(*I*)
graphite	*R*_int_ = 0.020
ω scans	θ_max_ = 27.0°, θ_min_ = 2.7°
Absorption correction: multi-scan (*SADABS*; Bruker, 1998)	*h* = −9→5
*T*_min_ = 0.942, *T*_max_ = 0.947	*k* = −11→10
6202 measured reflections	*l* = −18→18

### Refinement

**Table:**

Refinement on *F*^2^	Primary atom site location: structure-invariant direct methods
Least-squares matrix: full	Secondary atom site location: difference Fourier map
*R*[*F*^2^ > 2σ(*F*^2^)] = 0.043	Hydrogen site location: inferred from neighbouring sites
*wR*(*F*^2^) = 0.126	H-atom parameters constrained
*S* = 1.02	*w* = 1/[σ^2^(*F*_o_^2^) + (0.0675*P*)^2^ + 0.447*P*] where *P* = (*F*_o_^2^ + 2*F*_c_^2^)/3
2058 reflections	(Δ/σ)_max_ < 0.001
118 parameters	Δρ_max_ = 0.70 e Å^−^^3^
0 restraints	Δρ_min_ = −0.34 e Å^−^^3^

### Special details

**Table:**

Experimental. The compound was synthesized by following the procedure given in Shetty *et al.*, (2009)
Geometry. All e.s.d.'s (except the e.s.d. in the dihedral angle between two l.s. planes) are estimated using the full covariance matrix. The cell e.s.d.'s are taken into account individually in the estimation of e.s.d.'s in distances, angles and torsion angles; correlations between e.s.d.'s in cell parameters are only used when they are defined by crystal symmetry. An approximate (isotropic) treatment of cell e.s.d.'s is used for estimating e.s.d.'s involving l.s. planes.
Refinement. Refinement of *F*^2^ against ALL reflections. The weighted *R*-factor *wR* and goodness of fit *S* are based on *F*^2^, conventional *R*-factors *R* are based on *F*, with *F* set to zero for negative *F*^2^. The threshold expression of *F*^2^ > σ(*F*^2^) is used only for calculating *R*-factors(gt) *etc*. and is not relevant to the choice of reflections for refinement. *R*-factors based on *F*^2^ are statistically about twice as large as those based on *F*, and *R*- factors based on ALL data will be even larger.

### Fractional atomic coordinates and isotropic or equivalent isotropic displacement parameters (Å^2^)

**Table:**

	*x*	*y*	*z*	*U*_iso_*/*U*_eq_	
C1	−0.0414 (3)	1.0457 (2)	0.33340 (13)	0.0378 (4)	
C2	0.1087 (3)	0.7962 (2)	0.37011 (13)	0.0386 (4)	
C3	−0.0580 (3)	0.8166 (2)	0.41027 (13)	0.0391 (4)	
C4	−0.3211 (3)	1.0145 (3)	0.42024 (14)	0.0430 (5)	
H4A	−0.3036	1.0483	0.4830	0.052*	
H4B	−0.4087	0.9310	0.4176	0.052*	
C5	−0.3979 (3)	1.1460 (3)	0.3610 (2)	0.0664 (7)	
H5A	−0.4533	1.1044	0.3045	0.080*	
H5B	−0.4942	1.1960	0.3921	0.080*	
C6	−0.2579 (3)	1.2634 (3)	0.33910 (19)	0.0588 (6)	
H6A	−0.2179	1.3181	0.3943	0.071*	
H6B	−0.3147	1.3366	0.2964	0.071*	
C7	−0.0921 (3)	1.1943 (2)	0.29875 (14)	0.0444 (5)	
C8	−0.1417 (3)	0.9590 (2)	0.38916 (13)	0.0371 (4)	
C9	−0.1327 (3)	0.7027 (3)	0.46592 (16)	0.0494 (5)	
N1	0.2242 (3)	0.6773 (2)	0.37766 (13)	0.0539 (5)	
H1A	0.1994	0.6002	0.4108	0.065*	
H1B	0.3233	0.6781	0.3494	0.065*	
N2	−0.1907 (3)	0.6114 (3)	0.51076 (18)	0.0781 (7)	
O1	−0.0078 (2)	1.2613 (2)	0.24138 (12)	0.0622 (5)	
S1	0.15957 (6)	0.95344 (6)	0.30522 (3)	0.04133 (19)	

### Atomic displacement parameters (Å^2^)

**Table:**

	*U*^11^	*U*^22^	*U*^33^	*U*^12^	*U*^13^	*U*^23^
C1	0.0312 (9)	0.0402 (10)	0.0431 (10)	−0.0005 (8)	0.0107 (8)	0.0018 (8)
C2	0.0401 (10)	0.0374 (10)	0.0395 (10)	−0.0024 (8)	0.0107 (8)	0.0004 (8)
C3	0.0396 (10)	0.0402 (10)	0.0391 (10)	−0.0035 (8)	0.0126 (8)	0.0025 (8)
C4	0.0363 (10)	0.0507 (12)	0.0437 (10)	−0.0021 (9)	0.0147 (8)	−0.0008 (9)
C5	0.0440 (12)	0.0647 (16)	0.0931 (19)	0.0074 (12)	0.0246 (12)	0.0112 (14)
C6	0.0500 (13)	0.0545 (13)	0.0741 (16)	0.0128 (11)	0.0200 (12)	0.0146 (12)
C7	0.0387 (10)	0.0458 (12)	0.0499 (11)	0.0007 (9)	0.0107 (9)	0.0069 (9)
C8	0.0340 (9)	0.0421 (11)	0.0359 (9)	−0.0037 (8)	0.0080 (7)	−0.0024 (8)
C9	0.0490 (12)	0.0433 (12)	0.0585 (13)	0.0020 (9)	0.0211 (10)	0.0079 (10)
N1	0.0563 (11)	0.0431 (10)	0.0659 (12)	0.0097 (8)	0.0285 (9)	0.0104 (9)
N2	0.0778 (16)	0.0632 (14)	0.0985 (18)	0.0055 (13)	0.0416 (14)	0.0298 (14)
O1	0.0572 (10)	0.0555 (10)	0.0773 (11)	0.0072 (8)	0.0295 (8)	0.0249 (8)
S1	0.0344 (3)	0.0419 (3)	0.0496 (3)	−0.0004 (2)	0.0160 (2)	0.0072 (2)

### Geometric parameters (Å, °)

**Table:**

C1—C8	1.368 (3)	C4—H4B	0.9700
C1—C7	1.437 (3)	C5—C6	1.501 (3)
C1—S1	1.7504 (19)	C5—H5A	0.9700
C2—N1	1.339 (3)	C5—H5B	0.9700
C2—C3	1.402 (3)	C6—C7	1.512 (3)
C2—S1	1.732 (2)	C6—H6A	0.9700
C3—C8	1.414 (3)	C6—H6B	0.9700
C3—C9	1.424 (3)	C7—O1	1.230 (2)
C4—C8	1.499 (3)	C9—N2	1.138 (3)
C4—C5	1.526 (3)	N1—H1A	0.8600
C4—H4A	0.9700	N1—H1B	0.8600
			
C8—C1—C7	125.45 (18)	C4—C5—H5B	108.7
C8—C1—S1	112.37 (15)	H5A—C5—H5B	107.6
C7—C1—S1	122.18 (15)	C5—C6—C7	112.8 (2)
N1—C2—C3	128.63 (18)	C5—C6—H6A	109.0
N1—C2—S1	120.34 (15)	C7—C6—H6A	109.0
C3—C2—S1	111.02 (15)	C5—C6—H6B	109.0
C2—C3—C8	113.20 (17)	C7—C6—H6B	109.0
C2—C3—C9	122.26 (19)	H6A—C6—H6B	107.8
C8—C3—C9	124.54 (18)	O1—C7—C1	123.17 (19)
C8—C4—C5	111.26 (17)	O1—C7—C6	122.3 (2)
C8—C4—H4A	109.4	C1—C7—C6	114.57 (18)
C5—C4—H4A	109.4	C1—C8—C3	112.31 (17)
C8—C4—H4B	109.4	C1—C8—C4	121.39 (19)
C5—C4—H4B	109.4	C3—C8—C4	126.28 (17)
H4A—C4—H4B	108.0	N2—C9—C3	179.3 (3)
C6—C5—C4	114.3 (2)	C2—N1—H1A	120.0
C6—C5—H5A	108.7	C2—N1—H1B	120.0
C4—C5—H5A	108.7	H1A—N1—H1B	120.0
C6—C5—H5B	108.7	C2—S1—C1	91.10 (9)
			
N1—C2—C3—C8	178.3 (2)	C7—C1—C8—C4	0.0 (3)
S1—C2—C3—C8	−0.7 (2)	S1—C1—C8—C4	−178.70 (14)
N1—C2—C3—C9	−2.1 (4)	C2—C3—C8—C1	0.4 (3)
S1—C2—C3—C9	178.95 (17)	C9—C3—C8—C1	−179.2 (2)
C8—C4—C5—C6	−43.4 (3)	C2—C3—C8—C4	179.08 (18)
C4—C5—C6—C7	52.8 (3)	C9—C3—C8—C4	−0.5 (3)
C8—C1—C7—O1	−172.2 (2)	C5—C4—C8—C1	17.3 (3)
S1—C1—C7—O1	6.4 (3)	C5—C4—C8—C3	−161.3 (2)
C8—C1—C7—C6	8.3 (3)	C2—C3—C9—N2	38 (24)
S1—C1—C7—C6	−173.14 (17)	C8—C3—C9—N2	−142 (24)
C5—C6—C7—O1	146.5 (2)	N1—C2—S1—C1	−178.50 (18)
C5—C6—C7—C1	−33.9 (3)	C3—C2—S1—C1	0.57 (16)
C7—C1—C8—C3	178.76 (19)	C8—C1—S1—C2	−0.37 (17)
S1—C1—C8—C3	0.1 (2)	C7—C1—S1—C2	−179.11 (18)

### Hydrogen-bond geometry (Å, °)

**Table:**

*D*—H···*A*	*D*—H	H···*A*	*D*···*A*	*D*—H···*A*
N1—H1A···N2^i^	0.86	2.19	3.038 (3)	169
N1—H1B···O1^ii^	0.86	2.10	2.903 (3)	156
N1—H1B···S1^ii^	0.86	3.02	3.482 (2)	116

Symmetry codes: (i) −*x*, −*y*+1, −*z*+1; (ii) −*x*+1/2, *y*−1/2, −*z*+1/2.
